# Trends in Mortality from Diabetes and Other Non-Communicable Diseases Among Sri Lankan Adults: A Retrospective Population-Based Study, 2004–2020/2021

**DOI:** 10.3390/jcm14238568

**Published:** 2025-12-03

**Authors:** Harshana Munasinghe, Pansujee Dissanayaka, Mangalika Jayasundara, Mapa Prabhath Piyasena, Sobha Sivaprasad, Manjula D. Nugawela

**Affiliations:** 1 Department of Statistics & Computer Science, University of Kelaniya, Kelaniya 11600, Sri Lanka; pansujeed@kln.ac.lk (P.D.); jayasund@kln.ac.lk (M.J.); 2 Vision and Eye Research Institute, School of Medicine, Anglia Ruskin University, Cambridge CB1 1PT, UK; mapa.piyasena@aru.ac.uk; 3 National Institute of Health and Care Research Biomedical Research Centre, University College London (UCL) and Moorfields Eye Hospital, London EC1V 9EL, UK; sobha.sivaprasad@nhs.net; 4 Institute of Ophthalmology, University College London (UCL), 11-43 Bath St, London EC1V 9EL, UK; manjula.nugawela@ucl.ac.uk

**Keywords:** age-adjusted mortality, public health policy, Sri Lanka

## Abstract

**Background**: Non-Communicable Diseases (NCDs) are the leading cause of adult mortality worldwide, accounting for 74% of deaths. In Sri Lanka, assessing NCD mortality trends is essential for public health planning. This study evaluated trends in total and hospital-based NCD deaths from 2004 to 2020/2021, examining differences by disease category, age, and gender. **Methods**: Total NCD deaths (2004–2020; 2008 interpolated) were obtained from the Department of Census and Statistics, covering all registered deaths. Hospitalized deaths (2004–2021) were extracted from the Indoor Morbidity and Mortality Return (IMMR) dataset. Deaths were initially classified into nine major NCD categories based on ICD-11 codes and were subsequently consolidated into seven categories for the analysis. Age-adjusted mortality rates per 100,000 were calculated using the 2012 Sri Lankan Census. Percentage changes were assessed by age and gender, and trends tested using the Mann–Kendall method. **Results**: NCD mortality increased substantially, with differences between total and hospital deaths. Total deaths due to diabetes rose 169% and genitourinary causes 105%, whereas hospital deaths increased for the same condition by 34% and 123%, respectively. Women had higher total mortality, while men had higher hospital mortality across most NCDs. **Conclusions**: NCD deaths in Sri Lanka have risen over two decades, reflecting improved registration and gaps in early diagnosis and healthcare access. The greater increase in diabetes deaths outside hospital settings indicates possible under-diagnosis and suboptimal management of risk factors.

## 1. Introduction

According to the World Health Organization (WHO), non-communicable diseases (NCDs) account for 74% of all-cause mortality worldwide [1]. Globally, 42 million people die due to NCDs every year, especially in the working age population, resulting in a drain of human capital [1,2]. Therefore, the prevention of deaths due to noncommunicable diseases is essential for achieving a country’s Sustainable Development Goals.

NCDs include cancers, diabetes mellitus and endocrine disorders, neuropsychiatric conditions, sensory organ diseases, cardiovascular diseases, respiratory diseases, digestive diseases, genitourinary diseases, skin diseases and musculoskeletal diseases [3]. Among these, cardiovascular diseases result in the highest number of deaths (19 million), followed by cancers (10 million), chronic respiratory diseases (4 million) and diabetes mellitus and endocrine disorders (over 2 million) [1]. Together, these four disease categories account for 80% of all premature NCD deaths [1]. The mortality burden is projected to rise, especially in low- and middle-income countries (LMICs), where healthcare systems face significant challenges in early detection and chronic disease management [4,5].

In Sri Lanka, 83% of all deaths in 2019 were due to NCDs, with a 13.2% probability of dying from diabetes or endocrine causes between the ages of 30–70 years [1]. Modifiable lifestyle factors such as tobacco use, alcohol consumption, poor diet, and physical inactivity contribute significantly to the rising NCD burden [1]. Although the Sri Lankan government has prioritized NCD prevention through initiatives such as Healthy Lifestyle Centres established in 2011 [6,7,8], these remain underutilized. Moreover, a large proportion of deaths occur outside hospitals, only about 50.7% were hospital-reported in 2019 [9,10]. This disparity between hospital and non-hospital deaths suggests inequities in healthcare access, health-seeking behavior, and disease recognition [11].

Despite the growing NCD burden, there is a lack of comprehensive analysis on long-term national mortality trends in Sri Lanka, particularly comparing hospital and total mortality patterns. The only available study, published in 2018, examined premature NCD mortality from 2001 to 2010 [12], leaving a clear research gap regarding hospital versus non-hospital deaths.

Therefore, this study aimed to examine trends in NCD-related mortality in Sri Lanka from 2004 to 2020/2021, comparing hospital-based and total deaths across disease categories, age groups, and gender.

We hypothesize that, while overall NCD mortality has increased over time, hospital mortality trends vary by disease type and gender, reflecting disparities in healthcare access and utilization. Understanding these patterns is vital for informing public health policy, optimizing healthcare resource allocation, and guiding targeted interventions to reduce preventable NCD deaths in Sri Lanka.

## 2. Materials and Methods

### 2.1. Study Design

This was a retrospective descriptive trend analysis conducted to evaluate long-term patterns in mortality due NCDs in Sri Lanka. The study analyzed secondary data on total and hospital-based deaths between 2004 and 2020/2021.

### 2.2. Data Sources

Data for this analysis were extracted from multiple sources. The latest data on cause of death (total mortality data) were obtained from the Department of Census and Statistics Sri Lanka [13]. These data were available for the period from 2004 to 2020 (except 2008). The total mortality dataset compiled by the Department of Census and Statistics includes all registered deaths in Sri Lanka, encompassing both hospital and non-hospital deaths. Cause of death data for 2008 were not available at the Department of Census and Statistics Sri Lanka at the time of the analysis.

Data on hospitalized deaths due to NCDs in Sri Lanka (from 2004–2021) were extracted from the Indoor Morbidity and Mortality Return (IMMR) data available in the Ministry of Health Sri Lanka Open Data Portal [10]. The IMMR data consists of anonymized information on live discharges and hospitalized deaths in government hospitals of Sri Lanka categorized by gender, age and disease category [10]. People aged 17 years and above were included in this study.

### 2.3. Disease Classification

In our analysis, deaths due to NCDs were categorized using the International Classification of Diseases (ICD-11) codes. Initially, nine major disease groups were identified, including malignant neoplasms, diabetes mellitus and endocrine disorders, neuropsychiatric conditions, cardiovascular diseases, respiratory diseases, digestive diseases, genitourinary diseases, skin diseases and musculoskeletal diseases. However, due to the relatively low number of deaths recorded under neuropsychiatric conditions, skin diseases, and musculoskeletal diseases, these categories were combined and labeled as “Other”. Accordingly, seven major NCD groups were considered for the final analysis.

### 2.4. Statistical Analysis

Age-adjusted mortality rates per 100,000 population were calculated to account for variations in age and gender distribution across years. The direct standardization method was used, with age-adjusted mortality rates applied to the age distribution of a standard population. The 2012 Sri Lankan Census population, being the most recent national census, was used as the standard population for these calculations. This allowed meaningful comparison of mortality rates over time, independent of changes in the population age structure. Percentage increase in both total and hospital mortality was calculated.

The Mann–Kendall test was used to assess the significance of long-term trends in mortality rates. This non-parametric test is suitable because its test statistic is calculated directly without the need for simulation and it assumes the data points are independent and identically distributed. It was chosen as the observed mortality patterns were monotonic over time, with no evidence of abrupt changes or policy-driven structural shifts. As the cause of death data for 2008 were not available at the Department of Census and Statistics Sri Lanka at the time of the analysis, to maintain a continuous time series for trend analysis, the missing value for 2008 was estimated using linear interpolation between the preceding (2007) and following (2009) years. This interpolated value was used solely for constructing the linear regression trend line.

Hospital mortality rates and non-hospitalized mortality rates were compared using percentage increases. All the calculations were performed using R 4.4.2 software statistical significance was set at *p* < 0.05.

## 3. Results

### 3.1. Total NCD-Related Mortality

From 2004 to 2020, total deaths due to NCDs in Sri Lanka increased by 38%, with a significant rise observed among older age groups.

Cardiovascular diseases remained the leading cause of total NCD-related deaths throughout the study period. In 2004, the highest mortality rate was recorded for cardiovascular diseases (182 per 100,000). By 2020, cardiovascular diseases continued to be the primary cause of NCD mortality, followed by malignant neoplasms. Over this period, deaths due to cardiovascular diseases increased by 25%, while deaths from malignant neoplasms rose by 69%.

**Figure 1:**
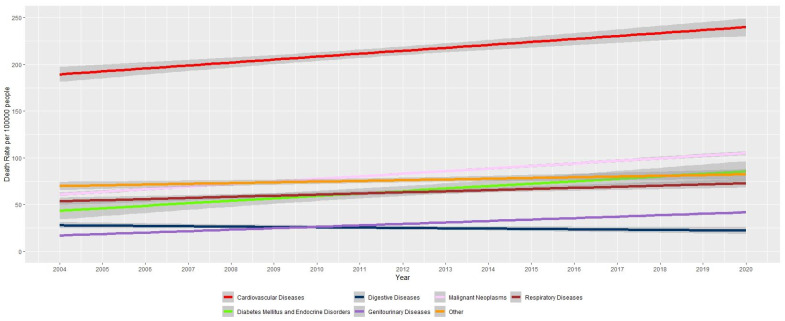
Age-adjusted total death rates due to non-communicable diseases in Sri Lanka.

As illustrated in Table 1 and Figure 1, mortality due to digestive showed a declining trend over time. The greatest percentage increase in total deaths was observed for diabetes mellitus and endocrine disorders with 169% increase.

**Figure 2:**
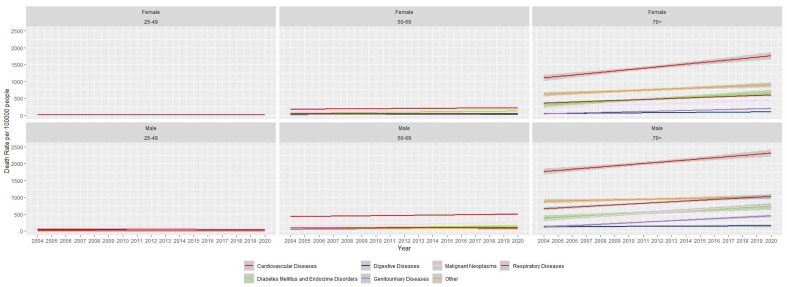
Age-adjusted total death rates due to non-communicable diseases in Sri Lanka by age and gender.

We further analyzed total mortality trends by age and gender, as illustrated in Figure 2 and Table A1. Higher death rates were observed among adults aged 70 years and above. Within this age group, cardiovascular diseases showed the highest increasing trend in both genders. Among individuals aged 70 years or older, deaths due to cardiovascular diseases increased by 34.1 (95% CI: 23.9–44.4) per 100,000 people per year in males and 40.8 (95% CI: 30.0–51.6) per 100,000 people per year in females. Similarly, an increasing trend was observed for diabetes mellitus and endocrine disorders in this age group, with deaths increasing by 23.3 (95% CI: 15.0–31.6) per 100,000 people per year in females and 21.3 (95% CI: 11.3–31.3) per 100,000 people per year in males. Overall, mortality rates among females were higher than those among males in this older age group.

In contrast, most mortality trends in the younger age group (25–49 years) were not statistically significant, and the few that were significant demonstrated a downward trend. Among individuals aged 50–69 years, cardiovascular diseases accounted for the highest increasing trend in mortality.

### 3.2. Results for Hospital Mortality

**Figure 3:**
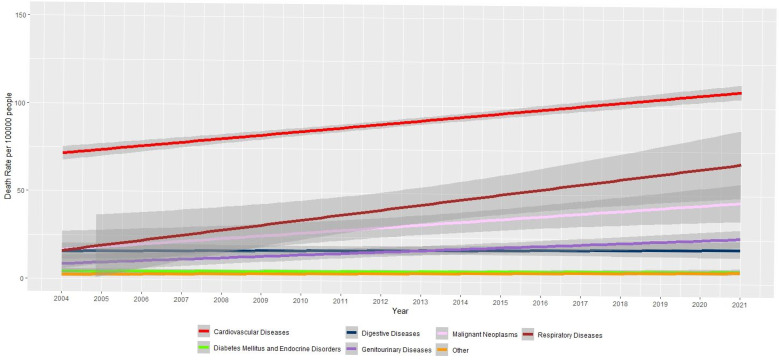
Hospitalized deaths due to non-communicable diseases in Sri Lanka.

Between 2004 and 2020, hospital deaths attributed to NCDs in Sri Lanka increased by 50%. As shown in Table 2 and Figure 3, the majority of hospital deaths were due to cardiovascular diseases (96 deaths per 100,000 in 2020) followed by respiratory diseases (43 deaths per 100,000 in 2020) and malignant neoplasms (33 deaths per 100,000 in 2020). Diabetes mellitus and endocrine disorders were among the top six causes of NCD-related hospital deaths, with rates increasing from 3.9 to 5.2 per 100,000, reflecting a 34% rise. Hospital deaths increased across all disease categories except for digestive diseases. In absolute terms, cardiovascular diseases remain the leading cause of hospital mortality. Although the “Other” disease category—which includes neuropsychiatric conditions, skin diseases, and musculoskeletal diseases showed a relatively high percentage increase, the absolute numbers remained low, rising from 2.6 per 100,000 deaths in 2004 to 7.2 per 100,000, deaths in 2020.

**Figure 4:**
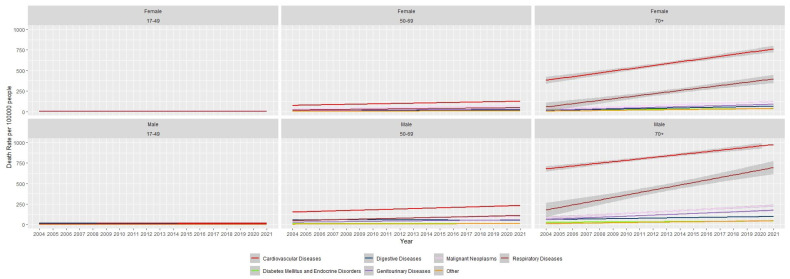
Age-adjusted hospitalized deaths due to non-communicable diseases in Sri Lanka by age and gender.

We further analyzed the trend by age and gender, as illustrated in Figure 4 and Table A2.

When examining trends by age and gender, NCDs showed a significant positive trend among individuals aged 70 years and above. In this age group, cardiovascular diseases exhibited the highest trend among females (22.5; 95% CI: 18.3–26.6 deaths per 100,000 per year), whereas respiratory diseases showed the highest trend among males (30.4; 95% CI: 22.1–38.6 deaths per 100,000 per year) (Table A2). Overall, most hospitalized death rates in older age groups were higher in males compared to females.

Among the younger age group (17–49 years), most trends were not statistically significant, including cardiovascular diseases, diabetes mellitus and endocrine disorders, genitourinary diseases in males, malignant neoplasms in males, musculoskeletal diseases, neuropsychiatric conditions in females, and respiratory diseases in males. Cardiovascular diseases showed the highest trend among individuals aged 50–60 years, in both genders.

### 3.3. Comparison of All Mortality, Hospital Mortality and Non-Hospital Mortality

As given in Table A3, non-hospitalized deaths were derived by subtracting hospital deaths from total deaths for each disease category. The proportion of all NCD deaths reported through the hospital system increased from 34% in 2004 to 37% in 2020. Correspondingly, the proportion of NCD deaths occurring outside hospitals declined from 66% to 63%, reflecting a gradual improvement in hospital reporting over time. When individual disease categories were examined, substantial increases were observed in non-hospitalized mortality across several NCDs. The most notable rise was seen for diabetes mellitus and endocrine disorders, with the non-hospitalized death rate increasing by 191%. Genitourinary diseases also showed a pronounced rise of 88% in non-hospitalized deaths, while cardiovascular and malignant neoplasm deaths increased more modestly, by 16% and 67%, respectively. In contrast, deaths due to digestive diseases declined slightly (−15%), and the “Other” category—which includes neuropsychiatric, skin, and musculoskeletal disorders remained largely unchanged (−3%).

## 4. Discussion

This study presents updated data on NCD-related mortality in Sri Lanka, revealing a concerning upward trend at the national level. The findings underscore the growing public health burden posed by NCDs, driven by factors such as delayed diagnosis, limited public awareness, and poor adherence to treatment protocols [1]. Elevated mortality rates among older age groups also suggest an increase in life expectancy over the past decade.

Notably, deaths due to cardiovascular diseases, respiratory illnesses, and malignant neoplasms have risen significantly, highlighting the urgent need to strengthen Sri Lanka’s health system. The sharp increase in cardiovascular-related deaths is particularly alarming, as many of these cases are preventable through improved primary care and enhanced patient education. Cancer emerged as the second leading cause of death during the study period, aligning with data from the National Cancer Control Programme, which reported a rise in cancer incidence from 27.9 to 52.3 per 100,000 population between 1985 and 2007 [14]. These trends emphasize the importance of implementing robust, community-based cancer screening initiatives. Deaths due to diabetes mellitus and endocrine disorders were one of the top six causes of deaths due to NCDs in Sri Lanka in the year 2020 and exhibited the highest increasing trend. This finding is consistent with the latest national studies on diabetes in Sri Lanka, which reported a significant rise in diabetes prevalence over the past decade [15,16]. This dramatic rise highlights the growing health burden posed by diabetes and endocrine disorders. This study also indicates that although the percentage of deaths due to diabetes mellitus and endocrine disorders reported in the hospital system has decreased over the years, there has been a sharp 191% increase in non-hospitalized deaths during the same period. On one hand, this trend may reflect improved outpatient and community-based management that helps prevent severe complications leading to hospital deaths [8]. However, several alternative explanations should be considered. First, inconsistencies in death certification practices may result in underreporting of diabetes as the underlying cause of death, since immediate causes such as cardiovascular or renal failure are often recorded instead [17]. Second, health-seeking behavior plays a role—individuals, particularly in rural or low-income settings, may delay or avoid hospital care due to financial, logistical, or cultural barriers [18]. Third, deficiencies in continuity and coordination of primary care may lead to inadequate monitoring, limited diagnostic capacity, limited availability of investigations and poor management with referral follow up for patients with diabetes complications [18]. Finally, the high prevalence of undiagnosed diabetes in the Sri Lankan population likely contributes to underestimation of hospital deaths, as individuals unaware of their diabetic status may succumb to complications without ever entering the formal healthcare system [16]. Together, these factors may explain the paradox of rising total diabetes-related mortality alongside declining hospital-reported deaths. This highlights the urgent need to strengthen diabetes screening and early detection, improve accuracy in cause-of-death certification, and enhance accessibility and continuity of diabetes care within the primary healthcare system. Another finding of our study was that the age-adjusted total death rates for NCDs in Sri Lanka are higher in females than males, while age-adjusted hospitalized death rates are higher in males than females. Potential explanations for this discrepancy include the under-utilization of hospital care by women, their relatively more sedentary lifestyles compared to men, and behavioral or cultural norms that prioritize healthcare for men, who are often the main financial providers in families [19,20,21]. Our study reveals the need to address this gender inequality in utilization of hospital care.

This study has many strengths. It represents the most recent analysis comparing trends in mortality rates of NCDs among adults in Sri Lanka. At the same time this is the first study which has compared overall deaths and total hospitalized deaths due to NCDs in Sri Lanka, providing a unique dual perspective on the burden of disease. By leveraging national administrative mortality data over an extended period, the study delivers valuable insights into evolving patterns and highlights critical areas for public health intervention and policy planning.

Despite these strengths, our study has several limitations. Due to data unavailability, we could not assess the relationship between mortality rates and other risk factors. Additionally, information on adults aged 17–25 years was limited. Specifically, trends in younger adults, particularly the 17–24-year age group, could not be examined separately. As we mentioned in methods, hospital mortality data were reported in broad age categories (17–49, 50–69, 70+ years), whereas population and total mortality data were available in narrower age bands (25–29, 30–34, etc.). To standardize age-specific rates for meaningful comparison, we aggregated hospital deaths into broader age categories and used corresponding aggregated population categories for total deaths. As a result, trends in the 17–24-year group are obscured, potentially masking early patterns of NCD risk development. Furthermore, as this study is based on aggregated national-level data, ecological bias cannot be excluded, and findings should not be interpreted as representing individual-level associations. Future research should aim to overcome these limitations through individual-level data linkage or longitudinal cohort studies to better elucidate causal pathways and age-specific risk trends.

## 5. Conclusions

In conclusion, both hospital and overall mortality due to noncommunicable diseases (NCDs) in Sri Lanka have demonstrated a rising trend between 2004 and 2020. This highlights the urgent need for comprehensive national strategies to mitigate modifiable risk factors such as unhealthy dietary habits, physical inactivity, and inadequate chronic disease management.

Future perspectives include strengthening national surveillance through integrated data systems that link hospital, community, and vital registration records to enable more accurate monitoring of NCD mortality trends. Expanding early detection and management programs within the primary healthcare system, supported by routine screening and continuity of care, will be critical to reducing premature mortality and improving population health outcomes.

For public health managers and policymakers, the findings highlight the need to prioritize investments in NCD prevention and control within existing health budgets. This can be achieved through sustainable health financing models, capacity building of primary healthcare teams, and ensuring equitable access to diagnostics, essential medicines, and long-term care.

## Figures and Tables

**Table 1:** Number of total deaths in 2004 and 2020.

Disease	Death Rate in 2004 (per 100,000)	Death Rate in 2020 (per 100,000)	Percentage Increase
Cardiovascular Diseases	181.5	227.4	25%
Respiratory Diseases	54.3	76.6	41%
Digestive Diseases	33.8	26.1	−23%
Malignant Neoplasms	59.8	101.2	69%
Genitourinary Diseases	16.2	33.1	105%
Diabetes Mellitus and Endocrine Disorders	27.7	74.5	169%
Other	73.0	75.3	3%

**Table 2:** Number of hospital deaths in 2004 and 2020.

Disease	Death Rate in 2004 (per 100,000)	Death Rate in 2020 (per 100,000)	Percentage Increase
Cardiovascular Diseases	67.8	96.1	42%
Respiratory Diseases	23.1	43.1	87%
Digestive Diseases	20.2	14.4	−28%
Malignant Neoplasms	19.2	33.4	74%
Genitourinary Diseases	7.7	17.3	123%
Diabetes Mellitus and Endocrine Disorders	3.9	5.2	34%
Other	2.6	7.2	178%

## Data Availability

The original data presented in the study are openly available in Ministry of Health Sri Lanka Open Data Portal at [https://www.health.gov.lk/annual-health-bulletin/. accessed on 20 October 2022] and Department of Census and Statistics Sri Lanka at [http://www.statistics.gov.lk/Population/StaticalInformation/VitalStatistics accessed on 10 October 2022].

## Abbreviations

The following abbreviations are used in this manuscript:

WHO	World Health Organization
NCD	Non-Communicable Diseases
IMMR	Indoor Morbidity and Mortality Return
ICD	International Classification of Diseases
MN	Malignant Neoplasms
DMED	Diabetes Mellitus and Endocrine Disorders
NC	Neuropsychiatric Conditions
CD	Cardiovascular Diseases
RD	Respiratory Diseases
DD	Digestive Diseases
SD	Skin Diseases
MD	Musculoskeletal Diseases
GD	Genitourinary Diseases

## Appendix A

**Table A1:** Results of the trend analysis of total death rates using the Mann–Kendall Test.

Disease	Gender	Age	Slope	95% CI		*p* Value of the Mann–Kendall Test
				Lower Limit	Upper Limit	
Cardiovascular Diseases	Female	17–49	0.06	−0.01	0.13	0.06
		50–69	2.94	2.38	3.51	<0.05
		>70	22.45	18.34	26.55	<0.05
	Male	17–49	0.07	−0.02	0.15	0.14
		50–69	4.67	3.76	5.59	<0.05
		>70	21.69	16.77	26.61	<0.05
Diabetes Mellitus and Endocrine Disorders	Female	17–49	−0.01	−0.02	0.01	0.24
		50–69	0.18	0.06	0.31	<0.05
		>70	0.83	0.51	1.15	<0.05
	Male	17–49	−0.02	−0.04	0.01	0.23
		50–69	0.11	−0.05	0.27	0.16
		>70	0.84	0.53	1.14	<0.05
Digestive Diseases	Female	17–49	−0.01	−0.03	0.00	<0.05
		50–69	0.70	0.61	0.78	<0.05
		>70	3.05	2.39	3.71	<0.05
	Male	17–49	−0.83	−1.08	−0.58	<0.05
		50–69	−0.05	−0.43	0.33	0.79
		>70	2.24	1.59	2.89	<0.05
Genitourinary Diseases	Female	17–49	0.04	0.01	0.07	<0.05
		50–69	1.23	1.12	1.35	<0.05
		>70	4.21	3.94	4.47	<0.05
	Male	17–49	−0.03	−0.09	0.02	0.39
		50–69	1.73	1.25	2.22	<0.05
		>70	6.54	5.55	7.54	<0.05
Malignant Neoplasms	Female	17–49	0.14	0.08	0.20	<0.05
		50–69	2.45	1.95	2.91	<0.05
		>70	5.29	4.54	6.04	<0.05
	Male	17–49	0.04	−0.03	0.11	0.24
		50–69	3.25	2.47	4.02	<0.05
		>70	8.64	7.15	10.12	<0.05
Respiratory Diseases	Female	17–49	0.07	0.01	0.15	<0.05
		50–69	1.74	1.33	2.14	<0.05
		>70	20.07	14.85	25.29	<0.05
	Male	17–49	−0.03	−0.11	0.05	0.43
		50–69	3.68	2.56	4.80	<0.05
		>70	30.36	22.10	38.63	<0.05
Other	Female	17–49	2.03	1.09	2.98	<0.05
		50–69	0.33	0.14	0.52	<0.05
		>70	1.89	1.08	2.69	<0.05
	Male	17–49	−0.01	−0.05	0.03	0.59
		50–69	0.42	0.20	0.64	<0.05
		>70	2.03	1.09	2.97	<0.05

**Table A2:** Results of trend analysis of hospitalized death rates using the Mann–Kendall Test.

Disease	Gender	Age	Slope	95% CI		*p* Value of the Mann–Kendall Test
				Lower Limit	Upper Limit	
Cardiovascular Diseases	Female	25–49	−0.25	−0.37	−0.14	<0.05
		50–69	2.68	1.82	3.55	<0.05
		>70	40.82	30.00	51.63	<0.05
	Male	25–49	−0.62	−1.21	−0.05	<0.05
		50–69	4.37	2.43	6.32	<0.05
		>70	34.11	23.85	44.35	<0.05
Diabetes Mellitus and Endocrine Disorders	Female	25–49	0.15	0.04	0.26	<0.05
		50–69	4.08	2.86	5.29	<0.05
		>70	23.30	15.02	31.57	<0.05
	Male	25–49	0.06	−0.16	0.28	0.69
		50–69	4.13	1.80	6.45	<0.05
		>70	21.33	11.34	31.33	<0.05
Digestive Diseases	Female	25–49	−0.10	−0.17	−0.03	<0.05
		50–69	0.67	0.36	0.98	<0.05
		>70	3.27	2.19	4.35	<0.05
	Male	25–49	−1.70	−2.99	−0.41	<0.05
		50–69	−1.31	−2.50	0.24	0.09
		>70	1.78	−2.76	6.34	0.41
Genitourinary Diseases	Female	25–49	0.02	−0.04	0.08	0.48
		50–69	1.86	1.53	2.20	<0.05
		>70	9.82	8.03	11.61	<0.05
	Male	25–49	−0.18	−0.94	0.58	0.61
		50–69	3.28	2.15	4.41	<0.05
		>70	20.59	16.24	24.94	<0.05
Malignant Neoplasms	Female	25–49	0.04	−0.07	0.15	0.49
		50–69	3.78	2.99	4.57	<0.05
		>70	15.18	13.70	16.65	<0.05
	Male	25–49	−0.01	−0.18	0.16	0.92
		50–69	7.43	6.42	8.43	<0.05
		>70	26.36	23.27	29.44	<0.05
Respiratory Diseases	Female	25–49	−0.12	−0.25	0.02	<0.05
		50–69	−0.05	−0.61	0.51	0.83
		>70	15.01	11.44	18.56	<0.05
	Male	25–49	−0.34	−0.51	−0.19	<0.05
		50–69	0.41	−0.55	1.38	0.36
		>70	22.77	16.22	28.93	<0.05
Other	Female	25–49	−0.08	−0.15	—0.02	<0.05
		50–69	0.66	0.38	0.93	<0.05
		>70	17.43	9.93	24.92	<0.05
	Male	25–49	−0.68	−1.00	−0.35	<0.05
		50–69	−0.26	−1.15	0.62	
		>70	8.35	0.88	15.82	<0.05

**Table A3:** Percentage increase comparison between hospitalized and non-hospitalized death rates.

	All Deaths		Hospital Deaths		Non Hospitalized Deaths		Hospitalized Death Rate (per 100000 People) (D = (B/x) * 100,000)		Non-Hospitalized Death Rate (per 100,000 People) (E = (C/x) * 100,000)		Percentage Increase for Hospitalized Death Rate	Percentage Increase for Non-Hospitalized Death Rate
	(A)		(B)		(C = A −B)							
	2004	2020	2004	2020	2004	2020	2004	2020	2004	2020		
Cardiovascular Diseases	26,440	33,135	9892	14,000	16,548	19,135	67.89	96.09	113.58	131.33	42%	16%
Diabetes Mellitus and Endocrine Disorders	4037	10,850	575	768	3462	10,082	3.95	5.27	23.76	69.20	34%	191%
Digestive Diseases	4931	3796	2941	2103	1990	1693	20.19	14.43	13.66	11.62	−28%	−15%
Genitourinary Diseases	2360	4834	1133	2525	1227	2309	7.78	17.33	8.42	15.85	123%	88%
Malignant Neoplasms	8714	14,752	2802	4870	5912	9882	19.23	33.42	40.58	67.82	74%	67%
Respiratory Diseases	7915	11,163	3363	6275	4552	4888	23.08	43.07	31.24	33.55	87%	7%
Other	10,636	10,966	379	1054	10,257	9912	2.60	7.23	70.40	68.03	178%	−3%

* x = Number of people in the population based on the 2012 national census data.
